# CYP2J2/EET reduces vulnerability to atrial fibrillation in chronic pressure overload mice

**DOI:** 10.1111/jcmm.14796

**Published:** 2019-11-20

**Authors:** Xuguang Li, Feng Zhu, Weidong Meng, Feng Zhang, Jiang Hong, Guobing Zhang, Fang Wang

**Affiliations:** ^1^ Department of Cardiology Shanghai General Hospital School of Medicine Shanghai Jiao Tong University Shanghai China

**Keywords:** atrial fibrillation, atrial fibrosis, epoxyeicosatrienoic acid, inflammation, Smad‐7

## Abstract

Growing evidence has well established the protective effects of CYP2J2/EET on the cardiovascular system. The aim of the present study was to determine whether CYP2J2/EET has a preventive effect on atrial fibrillation (AF) and to investigate the underlying mechanisms. Wild‐type mice were injected with or without AAV9‐CYP2J2 before abdominal aortic constriction (AAC) operation. After 8 weeks, compared with wild‐type mice, AAC mice display higher AF inducibility and longer AF durations, which were remarkably attenuated with AAV9‐CYP2J2. Also, AAV9‐CYP2J2 reduced atrial fibrosis area and the deposit of collagen‐I/III in AAC mice, accompanied by the blockade of TGF‐β/Smad‐2/3 signalling pathways, as well as the recovery in Smad‐7 expression. In vitro, isolated atrial fibroblasts were administrated with TGF‐β1, EET, EEZE, GW9662, SiRNA Smad‐7 and pre‐MiR‐21, and EET was demonstrated to restrain the differentiation of atrial fibroblasts largely dependent on Smad‐7, due to the inhibition of EET on MiR‐21. In addition, increased inflammatory cytokines, as well as activated NF‐κB pathways induced by AAC surgery, were also significantly blunted by AAV9‐CYP2J2 treatment. These effects of CYP2J2/EET were partially blocked by GW9662, the antagonist of PPAR‐γ. In conclusion, this study revealed that CYP2J2/EET ameliorates atrial fibrosis through modulating atrial fibroblasts activation by disinhibition of MiR‐21 on Smad‐7, and attenuates atrial inflammatory response by repressing NF‐κB pathways, reducing the vulnerability to AF, and CYP2J2/EET exerts its role at least partially through PPAR‐γ activation. Our findings might provide a novel upstream therapeutic strategy for AF.

## 1 INTRODUCTION

Atrial fibrillation (AF) is one of the most common arrhythmias seen clinically and is associated with a progressive increases in overall burden, incidence, prevalence, mortality and significant public health implications,^1^ yet current treatment paradigms have proven largely inadequate for important limitations, including adverse effect risks, incomplete efficacy and a significant long‐term recurrence rate.^2^ There is strong evidence supporting the involvement of fibrosis in the pathophysiology of AF, and development and progression of atrial fibrosis are the hallmark of structural remodelling in AF and are considered to be the substrate for AF perpetuation.^3^ Advanced atrial fibrosis is associated with more frequent paroxysms of AF, transformation of the arrhythmia into a permanent type and reduced effectiveness of anti‐arrhythmic therapy.^3^, ^4^ Atrial fibrosis causes intra‐ and inter‐atrial inhomogeneity in conduction, thus creating a substrate for local re‐entry and contributing to the maintenance and progression of AF. Besides that, emerging evidence indicated enhanced inflammatory responses in patients with AF and accompanied by increased circulating levels of pro‐inflammatory cytokines.^5^, ^6^ Further, NLRP3 inflammasome activation was recently shown to be involved in the pathogenesis of AF.^7^ Because AF is able to exacerbate inflammation and atrial fibrosis that further perpetuates the arrhythmia, reduction of inflammation and reversal of structural remodelling have increasingly become the focus of new therapeutic strategies for the prevention of AF.^3^, ^6^

CYP2J2 is a cytochrome P450 epoxygenase that is abundantly expressed in the human heart. CYP2J2 converts arachidonic acid to the four regioisomeric epoxyeicosatrienoic acids (5,6‐EET, 8,9‐EET, 11,12‐EET and 14,15‐EET), and EETs are further metabolized by the soluble epoxide hydrolase enzyme to form the corresponding 1,2‐diols, hydroxyeicosatetraenoic acids (DHET) with diminished bioactivity.^8^ EET is a pleiotropic cytokine that regulates a wide range of cellular actions and pathophysiological processes; in particular, the studies of EET in the cardiovascular system have become increasingly appreciated in recent years.^9^ Notably, CYP2J2/EET was recently demonstrated to prevent cardiac fibrosis.^10^, ^11^ Meanwhile, the anti‐inflammatory properties of CYP epoxygenase‐derived EET have been well evidenced 12, 13; further, we found that CYP2J2/EET inhibited TNF‐α‐induced cardiac injury by attenuation of nuclear factor‐kappaB activation.^14^ These protective effects might be mediated by peroxisome proliferator‐activated receptor gamma (PPAR‐γ), an important effector of EET.^15^ Due to the features of EET with anti‐fibrosis and anti‐inflammation, EETs derived from cytochrome P450 epoxygenase were speculated to have the potential to prevent AF. Westphal et al^16^ firstly reported that transgenic mice with cardiomyocyte‐specific overexpression of CYP2J2 enhanced cardiac EET biosynthesis, reducing ventricular tachyarrhythmia and AF susceptibility during maladaptive cardiac hypertrophy. Additionally, treatment with soluble epoxide hydrolase inhibitor (sEHI) by blocking the degradation of EET to DHET significantly reduces inflammation, oxidative stress, atrial structural and electric remodelling.^17^ However, the precise mechanisms of EET modulate AF remained obscure.

Here, we found that abdominal aortic constriction (AAC) operation enhanced the atrial fibrosis and inflammation response, resulting in increased AF occurrence and AF duration, whereas cardiac‐specific overexpression of CYP2J2 via adeno‐associated virus 9 (AAV9) vector reduced the susceptibility of AF, as well as ameliorated atrial fibrosis and inflammation in mice with AAC. Our study further elucidated that CYP2J2/EET exerts its effects by PPAR‐γ activation, thereby inhibiting atrial fibrosis through targeting MiR‐21/Smad‐7 and diminishing inflammatory response by inactivation of NFκB pathways, leading to the reduction in AF vulnerability.

## 2 METHODS

### 2.1 Ethics statement

All animal protocols in this study were approved by the Animal Care and Use Committee, School of Medicine, Shanghai Jiao Tong University, in accordance with the Guide for the Care and Use of Laboratory Animals published by the National Institutes of Health (Publication No. 85‐23, revised 1996) and National Standard of the People's Republic of China for Laboratory animal Guideline for ethical review of animal welfare. During the experiment, the general conditions of animals were observed daily, including food or water consumption, weight, infection and mortality. At the end of the experiment, the animals were killed using pentobarbital sodium.

### 2.2 Production of recombinant adeno‐associated virus 9

The rAAV vectors (Serotype 9) containing CYP2J2 or GFP were produced by triple plasmid cotransfection in HEK293 cells as previously described.^18^, ^19^, ^20^ The detailed description about the construction and preparation of the AAV vector is demonstrated in Figure S1. Purified rAAV9‐CYP2J2 (1 × 10^11^ pfu) was injected through tail vein 14 days prior to AAC operation. CYP2J2 expression and its metabolite EETs were measured Western blot and DHET kit according to previous methods,^21^ and the results are shown in Figure S2A,B.

### 2.3 Animal model

All mice were in the C57BL/6J background. The animal model of vulnerable AF was induced by pressure overload via AAC,^22^ and died mice resulted from operation will be excluded. Animal experiment 1: Eight‐week‐old male mice were randomly divided into 4 equal groups (10 mice/group): (a) WT mice undergoing a sham operation, (b) WT mice undergoing an AAC operation, (c) WT mice undergoing an AAC operation after AAV9‐CYP2J2 delivery for 14 days and (d) WT mice undergoing an AAC operation after AAV9‐GFP delivery for 14 days. Animal experiment 2: Eight‐week‐old male mice were randomly divided into four equal groups (6 mice/group): (a) WT mice undergoing a sham operation, (b) WT mice undergoing an AAC operation, (c) WT mice undergoing an AAC operation after AAV9‐CYP2J2 delivery for 14 days and (d) WT mice undergoing an AAC operation after AAV9‐CYP2J2 delivery for 14 days, and GW9662 was given a dose of 0.35 mg/kg per day in drinking water. After constriction with 8 weeks, all mice are killed and the left atriums of the heart were removed. All the mice were allowed free access to the regular chow and drinking water ad libitum*.*

### 2.4 Programmed electrical stimulation

Programmed electrical stimulation was performed according to previous study.^23^ Briefly, a transesophageal stimulation catheter (EPR‐800, Millar Instruments) was connected to a triggered stimulus isolation unit with variable current output. Inducibility of AF was tested by applying 2‐second bursts, through the automated stimulator. The first 2‐second burst had a cycle length (CL) of 40 ms, decreasing in each successive burst with a 2‐ms decrement down to a CL of 20 ms. AF was defined as a period of rapid irregular atrial rhythm lasting at least 2 seconds. If 2 or more bursts in the 3 series of bursts evoked an AF episode, AF was considered to be inducible in that animal.

### 2.5 Masson trichrome staining for collagen

Left atrial tissue samples were embedded in paraffin and sliced into 4‐µm‐thick sections, which underwent Masson's trichrome staining to highlight fibres. The percentage of fibrosis was measured as fibrosis areas/total given field areas × 100%.

### 2.6 Cell culture and treatment of atrial fibroblasts

Isolation and culture of mouse atrial fibroblasts was performed as previously described.^24^ These cultures contained N95% atrial fibroblasts as indicated by positive vimentin expression and negative sarcomeric actin expression. Atrial fibroblasts were treated with TGF‐β1 (10 ng/mL, Sigma), 11,12‐EET (1000 nmol L^−1^, Cayman), GW9662 (1000 nmol L^−1^, Sigma) and EEZE (1000 nmol L^−1^, Cayman) for 48 hours. SiRNA NC and SiRNA Smad‐7 (1000 nmol L^−1^, Santa Cruz) were transfected with siRNA transfection medium according to the instruction. Cells were transfected with either 20 nmol L^−1^ pre‐miR‐21 precursor or FAM3™ Dye‐Labeled pre‐miR miRNA precursor negative control #1 (Ambion), according to a previous study.^25^

### 2.7 α‐smooth muscle actin (α‐SMA) staining

α‐SMA staining was performed as described previously.^26^ Briefly, the cultured atrial fibroblasts were fixed with 4% PFA for 30 minutes, permeabilized with 0.3% Triton X‐100 for 30 minutes and blocked with 1% BSA for 60 minutes, and the cells were incubated with a primary antibody (α‐SMA) (1:200) overnight at 4°C. The cells were then washed and incubated with a rhodamine‐conjugated secondary antibody (1:500). The nuclei were stained with 4, 6‐diamidino‐2‐phenylindole (DAPI).

### 2.8 Real‐time PCR

Total RNA was extracted from cultured fibroblasts and left atriums using Trizol reagent (Invitrogen), according to the manufacturer's instructions. The expression of miR‐21 was analysed according to the instructions of the miRNAs qPCR Kit (Takara) using the ABI‐7900 Real‐Time PCR System. The bulge‐loop™ miRNA Primer Sets (one RT primer and a pair of qPCR primers) specific for miR‐21 and U6 were purchased from TSINGKE. The levels of miR‐21 were normalized to U6, respectively. U6 primer, forward: 5′‐CGCTTCGGCAGCACATATAC‐3′, reverse: 5′‐AAATATGGAACGCTTCACGA‐3′. MiR‐21 loop primer: 5′‐GTCGTATCCAGTGCAGGGTCCGAGGTATTCGCACTGGATACGACTCAACATC‐3′, F primer: 5′‐TGCGCTAGCTTATCAGACTGA‐3′, R primer: CCAGTGCAGGGTCCGAGGTATT.

### 2.9 Western blot

Western blot was performed as described previously.^27^ The cytoplasm protein and nuclear protein were extracted according to the instructions of the kit (Beyotime). The protein contents of extracts were determined using the Bradford method. Extracts were resolved using SDS‐PAGE and transferred to polyvinylidene difluoride filter membranes. Proteins were detected by immunoblotting and visualized using enhanced chemiluminescence. Antibodies (GAPDH, TGF‐β, Smad‐7, Smad‐2/3, p‐Smad‐2/3, Col‐I, Col‐III, α‐SMA, PPAR‐γ, NF‐κB, IκBα, p‐IκBα, TNF‐α, MCP‐1) were from Santa Cruz. The relative band densities were normalized against GAPDH.

### 2.10 Measurement of plasma cytokines

Plasma cytokines levels (TNF‐α, IL‐6, MCP‐1) are measured with ELISA kit (Jiancheng Bioengineering), according to the manufacturer's instructions.

### 2.11 Statistical analysis

All data are represented as mean ± SEM or percentage. Comparisons between two groups were analysed with unpaired *t* tests. The differences between multiple groups were performed by one‐way ANOVA analysis followed by a Newman‐Keuls test. A value of *P* < .05 was considered statistically significant.

## 3 RESULT

### 3.1 CYP epoxygenase 2J2 reduces the vulnerability to atrial fibrillation in mice with AAC

**Figure 1:**
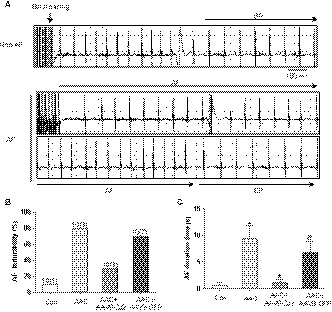
AAV9‐CYP2J2 inhibits atrial fibrillation inducibility and duration in mice with AAC. A, Representative simultaneous recordings of surface ECG in mice after programmed transesophageal burst pacing (arrows). B, Incidence of pacing‐induced AF in mice (n = 10/group). C, AF duration in mice(s) (n = 10/group). *P < .05, compare with control group; ^#^ *P* < .05, compare with AAC group; ^&^ *P* < .05, compare with AAC + CYP2J2 group. AF, atrial fibrillation; SR, sinus rhythm

To test whether CYP epoxygenase 2J2 is sufficient to reduce AF susceptibility, we first develop an AAV9 vector delivery cardio‐specific expression of CYP2J2. Then, mice injected with AAV9‐2J2 were performed AAC surgery, which exhibited a high inducibility of AF result from heart failure. Programmed electrical stimulation experiments were made at 8 weeks post‐AAC via transesophageal catheter to assess susceptibility to AF. Figure 1A shows a representative example of electrocardiogram after programmed electrical stimulation. AF occurred after the termination of the burst pacing and persisted more than 2 seconds, and the non‐induced AF mice still displayed sinus rhythm or AF persisted less than 2 seconds after the burst (Figure 1A). AF inducibility was higher in the mice with AAC (8/10, 80%) than in the sham mice (1/10, 10%, Figure 1B). Treatment with AAV9‐2J2 remarkably decreased the AF inducibility in AAC mice (3/10, 30%, Figure 1B), whereas AAV9‐GFP did not decline the higher inducibility of AF in AAC mice (7/10, 70%, Figure 1B). Additionally, the mean duration of the AF episode was longer in the mice with AAC than in the sham mice (0.62 ± 0.62s vs 9.363 ± 2.394s, *P* < .05; Figure 1C), whereas AAV9‐CYP2J2 treatment decreased the duration time of AF (1.108 ± 0.5902s). Accordingly, as a result of high AF inducibility, the mean duration time of AF in AAV9‐GFP‐treated mice is substantially more than the AAV9‐CYP2J2 group (6.703 ± 2.133s, *P* < .05; Figure 1C). These findings clearly suggest that CYP2J2 significantly reduces AF susceptibility in mice with AAC.

### 3.2 CYP epoxygenase 2J2 reduce atrial fibrosis in mice subject to AAC

**Figure 2:**
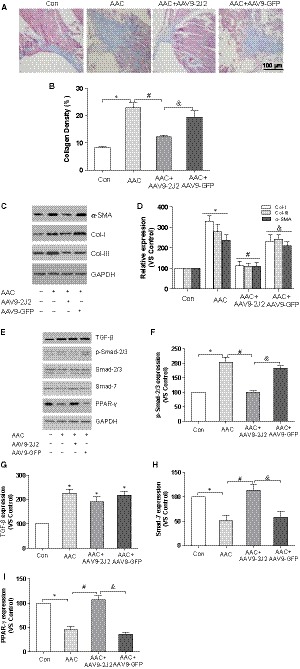
AAV9‐CYP2J2 reduces atrial fibrosis in mice with AAC. A, Representative images of left atrial (LA) fibrosis (Masson staining, which stains fibre blue, scale bar: 100 μm). B, Percentage of LA fibrosis, n = 5/group. C, Representative immunoblot of α‐SMA, collagen‐I and collagen‐III in left atria. D, Statistical analysis of relative expression of collagen‐I and collagen‐III, n = 3/group. E, Representative immunoblot of TGF‐β, Smad‐2/3 phosphorylation, Smad‐7 and PPAR‐γ in left atria. F, Statistical analysis of relative expression of Smad‐2/3 phosphorylation, n = 3/group. G, Statistical analysis of relative expression of TGF‐β, n = 3/group. H, Statistical analysis of relative expression of Smad‐7, n = 3/group. I, Statistical analysis of relative expression of PPAR‐γ, n = 3/group. Three independent experiments for Western blot, *P < .05, compare with control group; ^#^ *P* < .05, compare with AAC group; ^&^ *P* < .05, compare with AAC + CYP2J2 group

Next, we sought to explore the mechanisms that CYP2J2 prevents the vulnerability of AF. Thus, we measured interstitial fibrosis by Masson's staining in the left atria of wild‐type and AAC mice treated with AAV9‐2J2 or AAV9‐GFP. Summary data showed a marked increase in atrial interstitial fibrosis in AAC mice compared with sham mice (8.100 ± 0.6277% vs 22.74 ± 1.787%; *P* < .05; Figure 2A,B), whereas treatment with AAV9‐2J2 greatly reduced the percentage of fibrosis area (12.26 ± 0.5316%; *P* < .05; Figure 2A,B). In addition, we detected that increased deposition of α‐smooth muscle actin (α‐SMA) and collagen‐I and collagen‐III in the left atria of AAC mice were also mitigated by AAV9‐2J2 gene delivery (Figure 2C,D). To determine the mechanism that CYP2J2 inhibits atrial fibrosis, we further examined the fibrosis‐related TGF‐β/Smad pathways, and the data showed enhanced TGF‐β and phosphorylation of Smad‐2/3 with unchanged total Smad‐2/3 in the left atrium of mice with AAC surgery, whereas AAV9‐2J2 suppressed the phosphorylation of Smad‐2/3 (Figure 2E,F), without altering the expression of TGF‐β significantly (Figure 2E,G). Intriguingly, the expression of Smad‐7 was remarkably decreased in AAC mice, but AAV9‐2J2 treatment effectively restored Smad‐7 expression (Figure 2E,H). Similar to the previous study, reduced PPAR‐γ in the left atria of AAC mice was also rescued with AAV9‐2J2 (Figure 2E,I), suggesting that CYP2J2 attenuates the AAC‐induced atrial fibrosis by suppressing TGF‐β/Smad‐2/3 pathways via PPAR‐γ. In particular, Smad‐7 may be playing an important role in mediating the anti‐fibrotic action of CYP2J2/EET.

### 3.3 Epoxyeicosatrienoic acid suppress TGF‐β‐induced atrial fibroblasts activation

**Figure 3:**
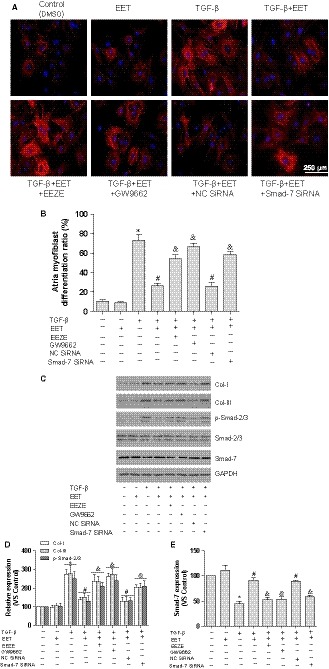
EET suppresses TGFβ1‐induced atrial fibroblasts activation. A, Representative images of atrial fibroblasts transdifferentiation with TGFβ1, EET, EEZE, GW9662 and SiRNA Smad‐7 (α‐SMA staining, scale bar: 250 μm). B, Statistical analysis of transdifferentiation ratio in atrial fibroblasts (10 visible areas/group; five independent experiments). C, Representative immunoblot of collagen‐I, collagen‐III, Smad‐2/3 phosphorylation and Smad‐7. D, Statistical analysis of relative expression of collagen‐I, collagen‐III and Smad‐2/3 phosphorylation, n = 3/group. E, Statistical analysis of relative expression of Smad‐7, n = 3/group. Three independent experiments for Western blot, *P < .05, compare with atrial fibroblasts without TGFβ1 stimulation; ^#^ *P* < .05, compare atrial fibroblasts with TGFβ1 stimulation; ^&^ *P* < .05, compare with atrial fibroblasts with TGFβ1 stimulation and EET treatment

In agreement with previous study,^10^, ^11^ AAV9‐2J2 improved the worsened cardiac function in AAC mice, as demonstrated in Figure S2C,D (heart weight/body weight ratio (HW/BW) and ejection fraction (EF)); therefore, the effects of CYP2J2 observed in the atria may be secondary to the improvement in cardiac function. To investigate the mechanisms that CYP2J2/EET inhibits atrial fibrosis and independent of cardiac function, and to assess the role of Smad‐7, the isolated atrial fibroblasts were treated with 11,12‐EET (1000 nmol L^−1^), 14,15‐EEZE (1000 nmol L^−1^, EET antagonist), GW9662 (1000 nmol L^−1^) and Smad‐7 SiRNA in the presence of TGF‐β1 (10 ng/mL), as TGF‐β1 is the most important profibrotic stimulators for cardiac fibroblasts. As a result, α‐SMA staining indicated that nearly 73.3% atrial fibroblasts were transdifferentiated into myofibroblasts with TGF‐β1 stimulation, which were dramatically abolished by EET treatment (26.7%, Figure 3A,B). Because myofibroblasts derive from cardiac fibroblasts and play a particularly significant role in cardiac fibrosis, they have a ∼2‐fold higher capacity to synthesize collagen. Indeed, TGF‐β exposure enhanced the synthesis of collagen‐I and collagen‐III from atrial fibroblasts/myofibroblasts, which were also largely ameliorated by the treatment with EET (Figure 3C,D). In the line with the fibrotic signalling in vivo, we found that the application of TGF‐β1 caused marked phosphorylation of Smad‐2/3 in atrial fibroblasts, whereas EET substantially repressed Smad‐2/3 phosphorylation (Figure 3C,D). Consistently, the reduction of Smad‐7 caused by TGF‐β stimulation was also recovered by EET treatment (Figure 3C,E). However, these effects of EET on transdifferentiation of atrial fibroblasts and TGF‐β/Smad pathways were blunted by pretreatment with EEZE, GW9662 and Smad‐7 SiRNA, respectively. Taken together, these results establish that EET suppresses the activity of atrial fibroblasts by inhibition of TGF‐β/Smad‐2/3 through Smad‐7, via PPAR‐γ activation.

### 3.4 CYP2J2/EET inhibits atrial fibrosis and atrial fibroblast activation by targeting MiR‐21/Smad‐7

**Figure 4:**
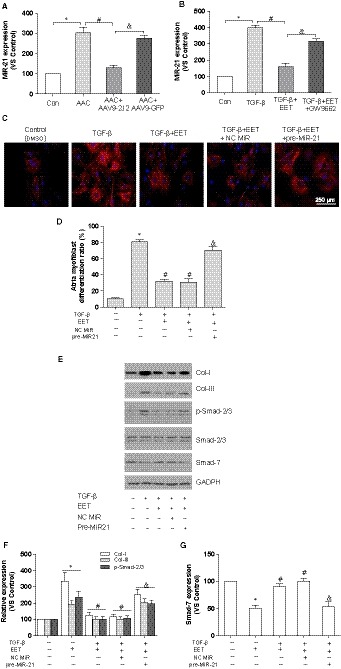
CYP2J2/EET inhibits atrial fibrosis and atrial fibroblasts activation by targeting MiR‐21/Smad‐7. A, The effects of AAV9‐CYP2J2 on MiR‐21 expression in mice with or without AAC; *P < .05, compare with control group; ^#^ *P* < .05, compare with AAC group; ^&^ *P* < .05, compare with AAC + CYP2J2 group, n = 10/group. B, The effects of EET on MiR‐21 expression in atrial fibroblasts with or without TGFβ1 stimulation, n = 5/group. C, Representative images of atrial fibroblasts transdifferentiation with TGFβ1, EET, pre‐MiR‐21 (α‐SMA staining, scale bar: 250 μm). D, Statistical analysis of transdifferentiation ratio in atrial fibroblasts (10 visible areas/group; five independent experiments). E, Representative immunoblot of collagen‐I, collagen‐III, Smad‐2/3 phosphorylation and Smad‐7. F, Statistical analysis of relative expression of collagen‐I, collagen‐III and Smad‐2/3 phosphorylation. G, Statistical analysis of relative expression of Smad‐7. Three independent experiments for Western blot, *P < .05, compare with atrial fibroblasts without TGFβ1 stimulation; ^#^ *P* < .05, compare atrial fibroblasts with TGFβ1 stimulation; ^&^ *P* < .05, compare with atrial fibroblasts with TGFβ1 stimulation and EET treatment

However, the link between CYP2J2/EET and Smad‐7 is less obvious. Importantly, increasing evidence supported Smad‐7 as the target of microRNA‐21 (MiR‐21), and MiR‐21 binds to 3′ terminus of Smad‐7 and then represses the expression of Smad‐7, contributing to cardiac fibrosis.^28^, ^29^ Because MiR‐21 was negatively regulated by PPAR‐γ,^30^ and PPAR‐γ was an important downstream effector of CYP2J2/EET,^10^ GW9662 abolished the increase in Smad‐7, and we speculated that CYP2J2/EET promotes Smad‐7 probably by modulating MiR‐21. Thus, we examined the expression of MiR‐21 in the left atria through real‐time PCR. As expected, the MiR‐21 expression level was 3‐fold higher in the left atria of AAC mice compared to sham and was remarkably declined in AAV9‐2J2‐treated mice (Figure 4A). Likewise, the TGF‐β‐treated atrial fibroblasts exhibited a higher MiR‐21 level compared to untreated atrial fibroblasts, whereas EET significantly decreased MiR‐21 level (Figure 4B). Similarly, the attenuation of EET on MiR‐21 was antagonized with GW9662. Next, to evaluate the role of MiR‐21, pre‐MiR‐21 (MiR‐21 precursor) was co‐treated with 11,12‐EET in the atrial fibroblasts, and α‐SMA staining demonstrated that the administration of pre‐MiR‐21 remarkably reversed the inhibitory effect of EET on transdifferentiation (Figure 4C,D). As such, we also tested collagen deposit and TGF‐β/Smad pathways, as shown in Figure 4E,F, and Col‐I, Col‐III and p‐Smad‐2/3 reduced by EET were restored with pre‐MiR‐21; in contrast, enhanced Smad‐7 in EET‐treated fibroblasts was down‐regulated by pre‐MiR‐21 treatment (Figure 4G). These results suggest that CYP2J2/EET might diminish atrial fibrosis by alleviating the suppression of MiR‐21 on Smad‐7 via PPAR‐γ.

### 3.5 CYP epoxygenase 2J2 inhibits atrial inflammations in mice with AAC

**Figure 5:**
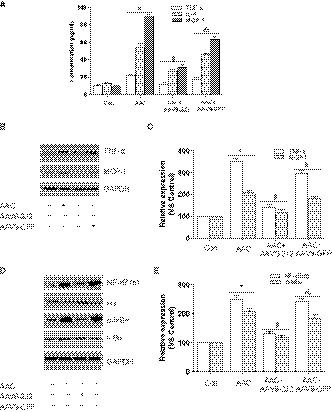
AAV9‐CYP2J2 inhibits atrial inflammations in mice with AAC. A, Serum concentration of cytokines (TNF‐α, IL‐6 and MCP‐1), n = 10/group. B, Representative immunoblot of TNF‐α and MCP‐1 in left atria. C, Statistical analysis of relative expression of TNF‐α and MCP‐1 in left atria, n = 3/group. D, Representative immunoblot of nuclear NFκB and IκBα phosphorylation in left atria. E, Statistical analysis of relative expression of nuclear NFκB and IκBα phosphorylation in left atria, n = 3/group. Three independent experiments for Western blot, *P < .05, compare with control group; ^#^ *P *< .05, compare with AAC group; ^&^ *P* < .05, compare with AAC + CYP2J2 group

It was well accepted that inflammation promotes the development and perpetuation of AF.^31^ Pro‐inflammatory cytokine levels were elevated with heart failure induced by TAC.^17^ Therefore, to evaluate whether CYP2J2/EET counteracts the inflammatory response in AAC mice, we measured important pro‐inflammatory cytokines and chemokines including TNF‐α, IL‐6 and MCP‐1, and our data demonstrate the serum level of TNF‐α, IL‐6 and MCP‐1 were significantly increased in mice with AAC (Figure 5A). In contrast, treatment with AAV9‐2J2 significantly decreased these cytokine levels. Besides that, we further found that enhanced expression of TNF‐α and MCP‐1 in the left atria of AAC mice, which were substantially attenuated by AAV9‐2J2 treatment (Figure 5B,C). Because NF‐κB represents one of the critical players in the cytokine‐mediated inflammation and is negatively regulated by CYP2J2/EET,^14^ we tested NF‐κB cascades in left atria of AAC mice. In the line with a previous study,^17^ AAC operation led to the phosphorylation of IkBα as well as increased nuclear translocation of NF‐κB, which were almost normalized by AAV9‐2J2 treatment (Figure 5B,C). These findings indicated that CYP2J2/EET lessens the inflammatory response in the left atria of AAC mice by inactivating NF‐κB pathways, relieving the development of AF substrate.

### 3.6 GW9662 blocks the effect of CYP 2J2 on atrial fibrillation in mice with AAC

**Figure 6:**
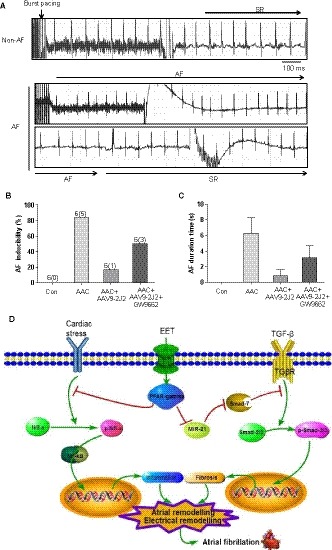
GW9662 blocked the inhibition of AAV9‐CYP2J2 on atrial fibrillation susceptibility in mice with AAC. A, Representative simultaneous recordings of surface ECG in mice after programmed transesophageal burst pacing (arrows). B, Incidence of pacing‐induced AF in mice (n = 6/group). C, AF duration in mice(s). AF, atrial fibrillation; SR, sinus rhythm. D, Summary of the possible mechanisms involved in the prevention of atrial fibrillation with CYP2J2/EET treatment

Finally, to verify the role of PPAR‐γ, GW9662, the PPAR‐γ antagonist, was co‐treated with AAV9‐2J2 in AAC mice, and programmed electrical stimulation experiments were performed to test the inducibility of AF. Figure 6A shows a representative example of electrocardiogram after programmed electrical stimulation. Similar to the above data, AF inducibility was higher in the mice with AAC (5/6, 83.3%) than in the sham mice (0/6, 0%, Figure 6B). Treatment with AAV9‐2J2 remarkably decreased the AF inducibility in AAC mice (1/6, 16.6%, Figure 6B), whereas GW9662 moderately abolished the effect of AAV9‐CYP2J2 in AAC mice (3/6, 50%, Figure 6B). Accordingly, the mean duration of the AF episode was shorter in the mice with AAV9‐2J2 than the AAC mice (0.800 ± 0.800s vs 6.300 ± 1.972s, n = 6, *P* < .05; Figure 6C), whereas GW9662 reversed the effect of AAV9‐2J2 (3.142 ± 1.519s, n = 6, *P* = .2025; Figure 6C), despite no statistical difference. Taken together, these data implicate that CYP2J2/EET exerts the inhibitory effect on AF partially via PPAR‐γ activation.

## 4 DISCUSSION

In the present study, we inquired whether EET‐induced alteration in atrial fibrosis and inflammation contributes to the prevention of AF. Hence, the major findings from our study include the following: (a) CYP2J2/EET reduces susceptibility to AF; (b) CYP2J2/EET reduces left atrial fibrosis by modulating atrial fibroblasts activation, and the mechanism by which EET activates PPAR‐γ eliminated the inhibitory effect of MiR‐21 on Smad‐7; (c) CYP2J2/EET inhibits inflammatory response in left atria by blocking NF‐κB pathways; and (d) these findings indicated that CYP2J2/EET prevents left atrial fibrosis by modulating atrial fibroblasts activity via regulating PPAR‐γ/MiR‐21/Smad‐7 pathway and ameliorated the inflammation by restraining NF‐κB pathways, leading to reduction of vulnerability to AF in mice with AAC. This study is potentially of great significance, because it unveils a novel feature to a prominent eicosanoid and suggests a new target for the prevention of AF.

Over decades, a large body of studies supported the pivotal role of the cytochrome P450 epoxygenase and its metabolites EET in the cardiovascular system,^8^ producing the hypothesis about anti‐arrhythmic potency of EET. Therefore, firstly, cardiac‐specific overexpression of CYP2J2 was shown to reduce the AF inducibility in pathologic cardiac hypertrophy, and the mechanism may be attributed to decreased atrial fibrosis.^16^ Thereafter, enhanced EET level with sEHI treatment was also showed to reduce atrial arrhythmia susceptibility in TAC mice, by antagonizing inflammatory response.^17^ Likewise, we observed the susceptibility of AF in AAC mice was significantly decreased with AAV9‐CYP2J2 gene delivery, confirming that CYP2J2/EET is sufficient to prevent AF.

A vulnerable substrate is necessary arrhythmogenic mechanism for AF, including the structural remodelling and electrical remodelling.^32^ Atrial fibrosis is considered to be the arrhythmogenic substrate for AF perpetuation and also emerges as an important pathophysiological contributor and link to AF recurrences and resistance to therapy.^3^ Fibrotic transformation of atrium causes the deterioration of atrial conduction, promoting re‐entry in the atrium, which may be directly related to the mechanisms responsible for maintaining AF.^33^ Those drugs that prevent the left atrial fibrosis demonstrated potent suppression in AF susceptibility,^3^ implying the efficiency of targeting at the fibrotic substrate in managing AF. Similar to a previous study,^16^ compared with AAC mice, the AAV9‐CYP2J2‐treated AAC mice exhibited a marked reduction in atrial fibrosis area and the deposition of collagen, and decreased AF susceptibility, which is also consistent with the effects of other anti‐fibrotic agents.^26^, ^34^ TGF‐β/Smad signalling pathway is mainly responsible for the regulation of fibrosis,^35^ because of negative feedback loop, Smad‐7 is decreased in response to TGF‐β or fibrotic stress, but Smad‐7 acts as a major inhibitory Smad protein, in turn, restrains fibrotic response through forming stable complexes with TGF‐β type I receptors and thereby blocking the phosphorylation of R‐Smads, or recruiting ubiquitin E3 ligases, leading to the ubiquitination and degradation of the activated type I receptors.^36^ Accordingly, rapid atrial pacing induces AF and fibrosis, associated with a marked increase in the expression of TGF‐β/p‐Smad2/3, but decreased Smad‐7 expression, and overexpression of Smad‐7 could blunt collagen synthesis.^37^ In addition, Smad‐7 gene transfer by a non‐invasive ultrasound microbubble was shown to reduce cardiac fibrosis via blocking TGF‐β/Smad‐3.^38^ Notably, TGF‐β1 transgenic mice exhibited a selective atrial fibrosis with increased vulnerability to AF.^23^ In this study, AAC operation induces atrial fibrosis with enhanced TGF‐β/Smad‐2/3 as well as declined Smad‐7; AAV9‐CYP2J2 treatment decreased Smad2/3 phosphorylation without altering TGF‐β, but restored Smad‐7 expression in AAC mice, implying that Smad‐7 might mediate the potency of CYP2J2/EET on atrial fibrosis.

The fibrosis is a complex process that involves increased production and activity of a variety of ECM proteins secreted by fibroblasts, and cardiac fibrosis is mainly dependent on fibroblasts. Fibroblasts constitute up to 75% of cardiac cells by number, when activated by profibrotic stimuli; fibroblasts differentiate into a secretory phenotype; and the myofibroblast, α‐SMA, is a hallmark of differentiation into the myofibroblast phenotype.^3^ AAV9‐CYP2J2 treatment also improved the cardiac function in mice with AAC. Thus, to clarify the effects of CYP2J2/EET on the atrial fibrosis independent of cardiac function, we tested the impact of EET on atrial fibroblasts activation with TGF‐β1 stimulation. Our findings demonstrate that the exposure of atrial fibroblasts to EET results in decreased transdifferentiation and collagen production upon TGF‐β1 stimulation and is associated with recovery of Smad‐7, leading to the defective activation of Smad‐2/3, but these actions of EET were largely abolished by SiRNA Smad‐7 and GW9662. Indeed, both PPAR‐γ and Smad‐7 have been displayed the capability of reducing atrial fibrosis and AF vulnerability,^37^, ^39^ and our data strongly favour that CYP2J2/EET suppresses atrial fibrosis by modulating atrial fibroblasts activation via PPAR‐γ/Smad‐7.

Emerging findings established Smad‐7 as the target of MiR‐21, and MiR‐21 represses the expression and transcript activity of Smad‐7 through binding the 3′ terminus of Smad‐7 mRNA, promoting the development of atrial fibrosis and AF.^28^, ^29^ Accumulating evidence indicates that the expression of miR‐21 is increased in atrial tissues and plasma samples from patients with AF, and MiR‐21 is an important non‐coding RNA that contributes to atrial fibrosis and AF 40, 41, 42; further, atrial injection with antagomir‐21 suppresses atrial fibrosis and reduces AF promotion in myocardial infarction model,^41^ confirming MiR‐21 an effective target for AF treatment. Specifically, MiR‐21 was shown to be negatively regulated by PPAR‐γ activator.^30^, ^43^ Resultantly, pre‐MiR‐21, the MiR‐21 precursor, effectively reversed the protective effects of EET on atrial fibroblasts exposed to TGF‐β1, including transdifferentiation, collagen production and Smad‐2/3 activation, whereas Smad‐7 expression was greatly declined with the application of pre‐MiR‐21. These results suggested that CYP2J2/EET reduces atrial fibrosis by disinhibition of MiR‐21 on Smad‐7 via activating PPAR‐γ in atrial fibroblasts, mitigating the AF substrate and the progression of AF.

Because EET was well known for exerting anti‐inflammation actions in the cardiovascular system, associated with inhibition of on NF‐κB cascade, a key signalling transduction of inflammation.^8^, ^14^ Meanwhile, growing evidence implies a significant role of inflammation in the pathogenesis of AF, including elevated serum levels of inflammatory biomarkers in AF subjects, the expression of inflammatory cytokines in cardiac tissues of AF patients and animal models of AF, and beneficial effects of anti‐inflammatory drugs in experimental AF paradigms.^6^ Moreover, heart failure impaired the atrial structure and enhanced inflammation response, promoting the initiation, progression and persistence of AF, which is one of the most common comorbidities leading to AF.^33^ In this study, we also observed AAC mice exhibited adverse cardiac function, along with elevated inflammatory cytokines, and AAV9‐CYP2J2 diminished AAC‐induced inflammatory cytokines levels while improving cardiac function. Similar to the previous study,^17^ the phosphorylation of IκBα and nuclear translocation of NF‐κB were also enhanced in left atria of AAC mice, which were significantly restrained by AAV9‐2J2 delivery, supporting that CYP2J2/EET reduces atrial inflammation by antagonizing NF‐κB pathways via activation of PPAR‐γ.

PPAR‐γ activator, pioglitazone, has been shown to attenuate atrial interstitial fibrosis and AF promotion in different animal models.^39^, ^44^, ^45^ More importantly, EET is viewed as an imagined ligand of PPAR‐γ,^15^, ^46^ and blocking PPAR‐γ can reverse the protection of EET on cardiac remodelling and inflammatory injury.^10^, ^14^ Therefore, in this study, GW9662 administration partially abolished the inhibitory effect of CYP2J2 on AF susceptibility, despite that AAV9‐CYP2J2 effectively restored the expression of PPAR‐γ in left atria of AAC mice, suggesting that PPAR‐γ, in part, mediates the action of CYP2J2/EET on AF.

In addition to pro‐inflammatory response, NF‐κB also plays an important role in cardiac hypertrophy,^10^, ^17^ which may increase AF occurrence. Moreover, overexpression of Smad‐7 was also demonstrated to restrain NF‐κB‐driven cardiac inflammation.^38^ Hence, the regulation of EET on NF‐κB may need further evaluation. Besides that, EET modulates the electrophysiological properties of the heart by regulating L‐type Ca^2+^, Na^+^ and ATP‐sensitive K^+^ (K_ATP_) channel activities,^46^, ^47^ which probably influence the electrical remodelling. Intriguingly, emerging evidence indicated that eicosapentaenoic acid (EPA, 20:5*n*‐3) and docosahexaenoic acid (DHA, 22:6*n*‐3) were also generated via the cytochrome P450 (CYP) epoxygenase pathway, and EPA/DHA were well known to possess anti‐inflammatory and anti‐arrhythmic properties and exert pleiotropic beneficial effects on cardiovascular function,^48^, ^49^ which may contribute to the inhibition of CYP2J2 on AF. Also, oxidative stress is viewed as an important inducer to atrial fibrosis and AF, and the modulation of EET on oxidative stress may be involved in suppressing AF.^10^ Furthermore, because of the limitations of the experimental AF model, and AAV9‐mediated CYP2J2 overexpression is not restricted to the atria, the reduction in AF susceptibility is inseparable from the blockade of CYP2J2/EET on cardiac remodelling, cardiac dysfunction and pro‐inflammatory cytokine formation. It should be noted that we only detected the effect of CYP2J2/EET on AF associated with heart failure induced by chronic overpressure via modulating atrial fibrosis and inflammation in the present study, and further studies are needed to verify our results in other animal models or AF patients.

Taken together, our data provide compelling evidence for the preventive effect of CYP2J2/EET on AF. Meanwhile, there are at least dual mechanisms mediated the inhibition of CYP2J2/EET on atrial fibrillation via activating PPAR‐γ (Figure 6D); on the one hand, CYP2J2/EET reduced atrial fibrosis through modulating atrial fibroblasts by eliminating the inhibition of MiR‐21 on Smad‐7; on the other hand, CYP2J2/EET ameliorated atrial inflammatory response by repressing NF‐κB pathways.

## CONFLICT OF INTEREST

The authors declare no conflict interests in this work.

## AUTHOR CONTRIBUTION

Xuguang Li and Fang Wang conceived and designed the study. Xuguang Li and Feng Zhu performed cell experiments and molecular biology experiments. Weidong Meng and Feng Zhang performed the animal experiments. Xuguang Li, Jiang Hong and Guobing Zhang analysed the data. Xuguang Li and Fang Wang wrote the manuscript. All authors read and approved the final manuscript.

## References

[1] Chugh SS , Havmoeller R , Narayanan K , et al. Worldwide epidemiology of atrial fibrillation: a Global Burden of Disease 2010 Study. Circulation. 2014;129:837‐847. 24345399 10.1161/CIRCULATIONAHA.113.005119 PMC4151302

[2] Heijman J , Algalarrondo V , Voigt N , et al. The value of basic research insights into atrial fibrillation mechanisms as a guide to therapeutic innovation: a critical analysis. Cardiovasc Res. 2016;109:467‐479. 26705366 10.1093/cvr/cvv275 PMC4777910

[3] Nattel S . Molecular and cellular mechanisms of atrial fibrosis in atrial fibrillation. JACC Clin Electrophysiol. 2017;3:425‐435. 29759598 10.1016/j.jacep.2017.03.002

[4] Dzeshka MS , Lip GY , Snezhitskiy V , et al. Cardiac fibrosis in patients with atrial fibrillation: mechanisms and clinical implications. J Am Coll Cardiol. 2015;66:943‐959. 26293766 10.1016/j.jacc.2015.06.1313

[5] Guo Y , Lip GY , Apostolakis S . Inflammation in atrial fibrillation. J Am Coll Cardiol. 2012;60:2263‐2270. 23194937 10.1016/j.jacc.2012.04.063

[6] Harada M , Van Wagoner DR , Nattel S . Role of inflammation in atrial fibrillation pathophysiology and management. Circ J. 2015;79:495‐502. 25746525 10.1253/circj.CJ-15-0138 PMC4457364

[7] Yao C , Veleva T , Scott L Jr , et al. Enhanced cardiomyocyte NLRP3 inflammasome signaling promotes atrial fibrillation. Circulation. 2018;138:2227‐2242. 29802206 10.1161/CIRCULATIONAHA.118.035202 PMC6252285

[8] Xu X , Zhang XA , Wang DW . The roles of CYP450 epoxygenases and metabolites, epoxyeicosatrienoic acids, in cardiovascular and malignant diseases. Adv Drug Deliv Rev. 2011;63:597‐609. 21477627 10.1016/j.addr.2011.03.006

[9] Imig JD . Prospective for cytochrome P450 epoxygenase cardiovascular and renal therapeutics. Pharmacol Ther. 2018;192:1‐19. 29964123 10.1016/j.pharmthera.2018.06.015 PMC6263841

[10] He Z , Zhang X , Chen C , et al. Cardiomyocyte‐specific expression of CYP2J2 prevents development of cardiac remodelling induced by angiotensin II. Cardiovasc Res. 2015;105:304‐317. 25618409 10.1093/cvr/cvv018 PMC4351370

[11] Zhou C , Huang J , Li Q , et al. CYP2J2‐derived EETs attenuated ethanol‐induced myocardial dysfunction through inducing autophagy and reducing apoptosis. Free Radic Biol Med. 2018;117:168‐179. 29427791 10.1016/j.freeradbiomed.2018.02.009

[12] Deng Y , Theken KN , Lee CR . Cytochrome P450 epoxygenases, soluble epoxide hydrolase, and the regulation of cardiovascular inflammation. J Mol Cell Cardiol. 2010;48:331‐341. 19891972 10.1016/j.yjmcc.2009.10.022 PMC2813356

[13] Node K , Huo Y , Ruan X , et al. Anti‐inflammatory properties of cytochrome P450 epoxygenase‐derived eicosanoids. Science. 1999;285:1276‐1279. 10455056 10.1126/science.285.5431.1276 PMC2720027

[14] Zhao G , Wang J , Xu X , et al. Epoxyeicosatrienoic acids protect rat hearts against tumor necrosis factor‐alpha‐induced injury. J Lipid Res. 2012;53:456‐466. 22223859 10.1194/jlr.M017319 PMC3276469

[15] Liu Y , Zhang Y , Schmelzer K , et al. The antiinflammatory effect of laminar flow: the role of PPARgamma, epoxyeicosatrienoic acids, and soluble epoxide hydrolase. Proc Natl Acad Sci USA. 2005;102:16747‐16752. 16267130 10.1073/pnas.0508081102 PMC1276614

[16] Westphal C , Spallek B , Konkel A , et al. CYP2J2 overexpression protects against arrhythmia susceptibility in cardiac hypertrophy. PLoS ONE ONE. 2013;8:e73490. 10.1371/journal.pone.0073490 PMC3758319 24023684

[17] Sirish P , Li N , Timofeyev V , et al. Molecular mechanisms and new treatment paradigm for atrial fibrillation. Circ Arrhythm Electrophysiol. 2016;9:e003721. 27162031 10.1161/CIRCEP.115.003721 PMC4869994

[18] Li XG , Yan JT , Xu XZ , et al. Recombinant adeno‐associated virus‐mediated delivery of antisense angiotensin II receptor 1 gene attenuates hypertension development. Acta Pharmacol Sin. 2007;28:1737‐1745. 17959024 10.1111/j.1745-7254.2007.00676.x

[19] Wang T , Li H , Zhao C , et al. Recombinant adeno‐associated virus‐mediated kallikrein gene therapy reduces hypertension and attenuates its cardiovascular injuries. Gene Ther. 2004;11:1342‐1350. 15175642 10.1038/sj.gt.3302294

[20] Xiao X , Li J , Samulski RJ . Production of high‐titer recombinant adeno‐associated virus vectors in the absence of helper adenovirus. J Virol. 1998;72:2224‐2232. 9499080 10.1128/jvi.72.3.2224-2232.1998 PMC109519

[21] Xiao B , Li X , Yan J , et al. Overexpression of cytochrome P450 epoxygenases prevents development of hypertension in spontaneously hypertensive rats by enhancing atrial natriuretic peptide. J Pharmacol Exp Ther. 2010;334:784‐794. 20501636 10.1124/jpet.110.167510 PMC2939659

[22] Wang Q , Chen Y , Zhang D , et al. Activin receptor‐like kinase 4 haplodeficiency mitigates arrhythmogenic atrial remodeling and vulnerability to atrial fibrillation in cardiac pathological hypertrophy. J Am Heart Assoc. 2018;7:e008842. 30369314 10.1161/JAHA.118.008842 PMC6201394

[23] Verheule S , Sato T , Tt E , et al. Increased vulnerability to atrial fibrillation in transgenic mice with selective atrial fibrosis caused by overexpression of TGF‐beta1. Circ Res. 2004;94:1458‐1465. 15117823 10.1161/01.RES.0000129579.59664.9d PMC2129102

[24] Tsai CT , Tseng CD , Hwang JJ , et al. Tachycardia of atrial myocytes induces collagen expression in atrial fibroblasts through transforming growth factor beta1. Cardiovasc Res. 2011;89:805‐815. 21134900 10.1093/cvr/cvq322

[25] Weber M , Baker MB , Moore JP , et al. MiR‐21 is induced in endothelial cells by shear stress and modulates apoptosis and eNOS activity. Biochem Biophys Res Commun. 2010;393:643‐648. 20153722 10.1016/j.bbrc.2010.02.045 PMC3717387

[26] Ma S , Ma J , Guo L , et al. Tongguan capsule‐derived herb reduces susceptibility to atrial fibrillation by inhibiting left atrial fibrosis via modulating cardiac fibroblasts. J Cell Mol Med. 2019;23:1197‐1210. 30456908 10.1111/jcmm.14022 PMC6349173

[27] Li X , Zhao G , Ma B , et al. 20‐Hydroxyeicosatetraenoic acid impairs endothelial insulin signaling by inducing phosphorylation of the insulin receptor substrate‐1 at Ser616. PLoS ONE ONE. 2014;9:e95841. 10.1371/journal.pone.0095841 PMC3998975 24763529

[28] He X , Zhang K , Gao X , et al. Rapid atrial pacing induces myocardial fibrosis by down‐regulating Smad7 via microRNA‐21 in rabbit. Heart Vessels. 2016;31:1696‐1708. 26968995 10.1007/s00380-016-0808-z PMC5043001

[29] Yuan J , Chen H , Ge D , et al. Mir‐21 promotes cardiac fibrosis after myocardial infarction via targeting Smad7. Cell Physiol Biochem. 2017;42:2207‐2219. 28817807 10.1159/000479995

[30] Green DE , Murphy TC , Kang BY , et al. Peroxisome proliferator‐activated receptor‐gamma enhances human pulmonary artery smooth muscle cell apoptosis through microRNA‐21 and programmed cell death 4. Am J Physiol Lung Cell Mol Physiol. 2017;313:L371‐L383. 28522568 10.1152/ajplung.00532.2016 PMC5582937

[31] Scott L Jr , Li N , Dobrev D . Role of inflammatory signaling in atrial fibrillation. Int J Cardiol. 2019;287:195‐200. 30316645 10.1016/j.ijcard.2018.10.020 PMC6447485

[32] Kirchhof P , Benussi S , Kotecha D , et al. 2016 ESC Guidelines for the management of atrial fibrillation developed in collaboration with EACTS. Eur Heart J. 2016;37:2893‐2962. 27567408 10.1093/eurheartj/ehw210

[33] Heijman J , Voigt N , Nattel S , et al. Cellular and molecular electrophysiology of atrial fibrillation initiation, maintenance, and progression. Circ Res. 2014;114:1483‐1499. 24763466 10.1161/CIRCRESAHA.114.302226

[34] Kume O , Takahashi N , Wakisaka O , et al. Pioglitazone attenuates inflammatory atrial fibrosis and vulnerability to atrial fibrillation induced by pressure overload in rats. Heart Rhythm. 2011;8:278‐285. 21034856 10.1016/j.hrthm.2010.10.029

[35] Yan X , Xiong X , Chen YG . Feedback regulation of TGF‐beta signaling. Acta Biochim Biophys Sin (Shanghai). 2018;50:37‐50. 29228156 10.1093/abbs/gmx129

[36] Yan X , Liu Z , Chen Y . Regulation of TGF‐beta signaling by Smad7. Acta Biochim Biophys Sin (Shanghai). 2009;41:263‐272. 19352540 10.1093/abbs/gmp018 PMC7110000

[37] He X , Gao X , Peng L , et al. Atrial fibrillation induces myocardial fibrosis through angiotensin II type 1 receptor‐specific Arkadia‐mediated downregulation of Smad7. Circ Res. 2011;108:164‐175. 21127293 10.1161/CIRCRESAHA.110.234369 PMC3035429

[38] Wei LH , Huang XR , Zhang Y , et al. Smad7 inhibits angiotensin II‐induced hypertensive cardiac remodelling. Cardiovasc Res. 2013;99:665‐673. 23761400 10.1093/cvr/cvt151

[39] Shimano M , Tsuji Y , Inden Y , et al. Pioglitazone, a peroxisome proliferator‐activated receptor‐gamma activator, attenuates atrial fibrosis and atrial fibrillation promotion in rabbits with congestive heart failure. Heart Rhythm. 2008;5:451‐459. 18313605 10.1016/j.hrthm.2007.12.010

[40] Adam O , Lohfelm B , Thum T , et al. Role of miR‐21 in the pathogenesis of atrial fibrosis. Basic Res Cardiol. 2012;107:278. 22760500 10.1007/s00395-012-0278-0

[41] Cardin S , Guasch E , Luo X , et al. Role for MicroRNA‐21 in atrial profibrillatory fibrotic remodeling associated with experimental postinfarction heart failure. Circ Arrhythm Electrophysiol. 2012;5:1027‐1035. 22923342 10.1161/CIRCEP.112.973214

[42] Huang Z , Chen XJ , Qian C , et al. Signal transducer and activator of transcription 3/MicroRNA‐21 feedback loop contributes to atrial fibrillation by promoting atrial fibrosis in a rat sterile pericarditis model. Circ Arrhythm Electrophysiol. 2016;9:e003396. 27406600 10.1161/CIRCEP.115.003396 PMC4956678

[43] Green DE , Murphy TC , Kang BY , et al. PPARgamma ligands attenuate hypoxia‐induced proliferation in human pulmonary artery smooth muscle cells through modulation of MicroRNA‐21. PLoS ONE ONE. 2015;10:e0133391. 10.1371/journal.pone.0133391 PMC4514882 26208095

[44] Xu D , Murakoshi N , Igarashi M , et al. PPAR‐gamma activator pioglitazone prevents age‐related atrial fibrillation susceptibility by improving antioxidant capacity and reducing apoptosis in a rat model. J Cardiovasc Electrophysiol. 2012;23:209‐217. 21954843 10.1111/j.1540-8167.2011.02186.x

[45] Gu J , Liu X , Wang QX , et al. Beneficial effects of pioglitazone on atrial structural and electrical remodeling in vitro cellular models. J Mol Cell Cardiol. 2013;65:1‐8. 24100253 10.1016/j.yjmcc.2013.09.016

[46] Spector AA . Arachidonic acid cytochrome P450 epoxygenase pathway. J Lipid Res. 2009;50(Suppl):S52‐56. 18952572 10.1194/jlr.R800038-JLR200 PMC2674692

[47] Ke Q , Xiao YF , Bradbury JA , et al. Electrophysiological properties of cardiomyocytes isolated from CYP2J2 transgenic mice. Mol Pharmacol. 2007;72:1063‐1073. 17652182 10.1124/mol.107.035881 PMC2243182

[48] Arnold C , Markovic M , Blossey K , et al. Arachidonic acid‐metabolizing cytochrome P450 enzymes are targets of {omega}‐3 fatty acids. J Biol Chem. 2010;285:32720‐32733. 20732876 10.1074/jbc.M110.118406 PMC2963419

[49] Schunck WH , Konkel A , Fischer R , et al. Therapeutic potential of omega‐3 fatty acid‐derived epoxyeicosanoids in cardiovascular and inflammatory diseases. Pharmacol Ther. 2018;183:177‐204. 29080699 10.1016/j.pharmthera.2017.10.016

