# Supplementary material for: CYP2J2/EET reduces vulnerability to atrial fibrillation in chronic pressure overload mice
Source: J Cell Mol Med. 2019 Nov 20;24(1):862–74. doi: 10.1111/jcmm.14796 (PMC6933320; doi:10.1111/jcmm.14796)
Supplement: Supplementary file 1 [file JCMM-24-862-s001.pdf]

Figure S1

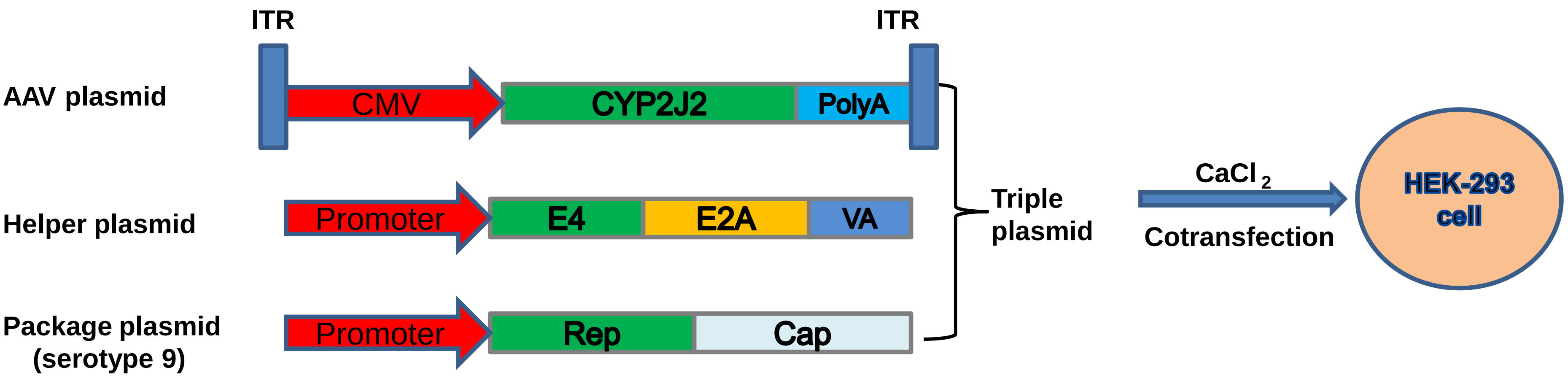

Figure S1: Production of recombinant adeno-associated virus 9: The rAAV-CYP2J2 and rAAV-GFP vector plasmids were constructed by inserting the human CYP2J2 cDNA and GFP cDNA into the AAV vector driven by the cytomegalovirus promoter at BamH I and Not I sites, respectively. The vectors for rAAV-CYP2J2 and rAAV-GFP were prepared using a triple plasmid cotransfection method in 293 cell lines. Human 293 cells were grown in Dulbecco's modified Eagle medium (DMEM) supplemented with 10% fetal bovine serum (FBS) and antibiotics in a 5% CO_2 atmosphere at 37℃. Immediately before transfection, each 15-cm-diameter plate of the cells (70-80% confluent) was fed with 15 ml of fresh DMEM containing 10% fetal bovine serum. A total of 50 μg plasmid DNA per 15 cm plate (AAV-CYP2J2 or AAV-GFP vector plasmids/package plasmid /helper plasmid were 1:1:1 molar ratios was dissolved in 1 ml of 0.25 M $CaCl_2$ and then quickly mixed with 1 ml of HEPES-buffered saline (50 mM HEPES, 280 mM NaCl, 1.5 mM Na_2HPO_4 (PH 7.1-7.2) and added to the cells. At 8-12 h after transfection, the medium was replaced with fresh DMEM containing 10% FBS. The cells were harvested at 48-72 h post transfection. After low-speed centrifugation on a tabletop centrifuge, the cell pellets were resuspended in 100 mM NaCl, 10 mM Tris–HCl (pH 8.0) and subjected to three cycles of freeze-thaw, and cell debris was removed by centrifugation. AAV vectors were purified with CsCl density gradient purification. The eluted rAAV was aliquoted and stored at -80 ℃.

Figure S2

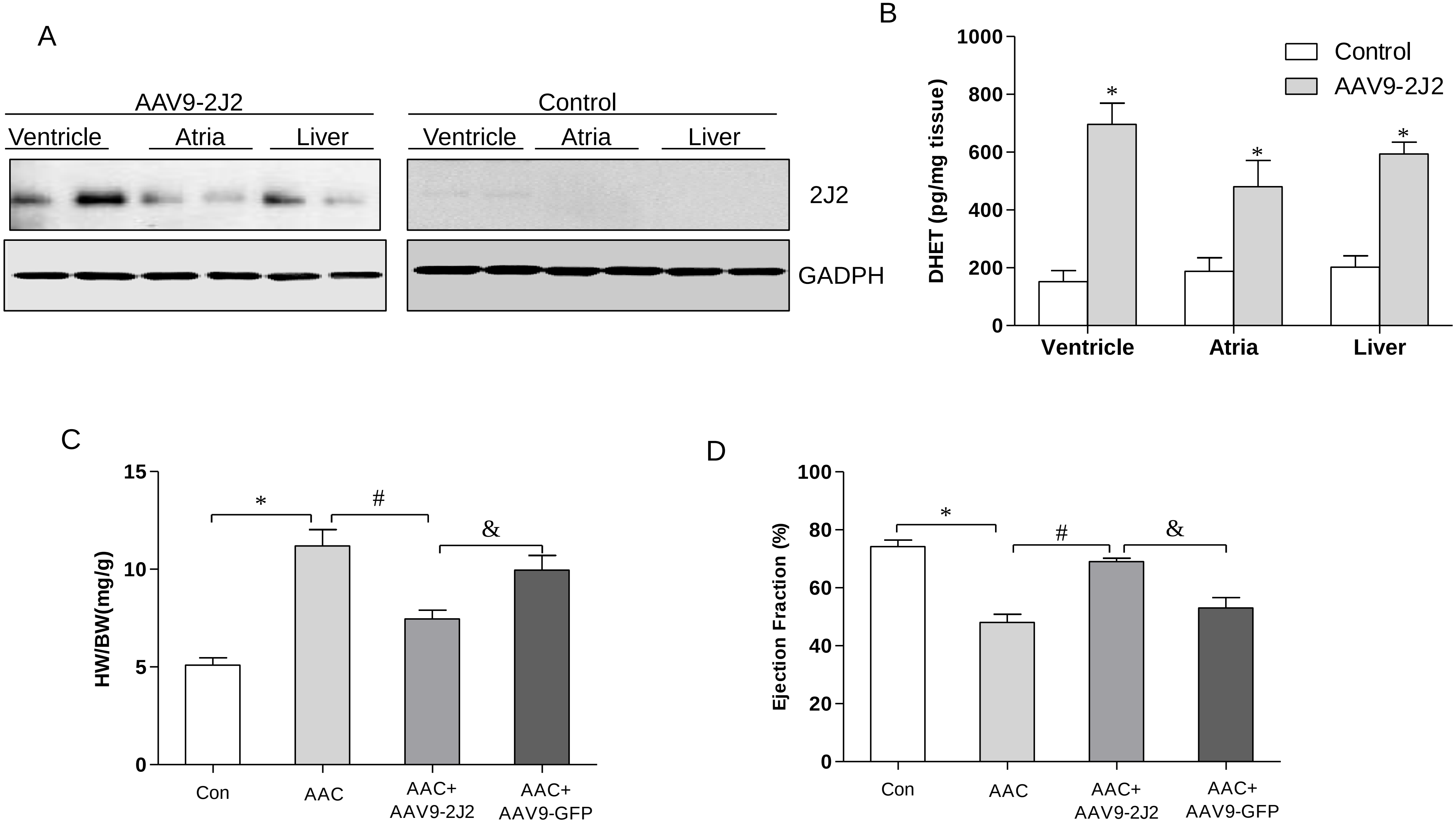

FigureS2: (A) Representative Western blots depicting expression of CYP2J2 in atria, ventricle and liver of mice with or without AAV9-2J2; (B) Analyses of DHET level in ventricle, atria and liver of mice with or without AAV9-2J2; (C) Analyses of heart weight/body weight ratio (HW/BW) in mice; (D)) Analyses of ejection fraction (EF). *: $p<0.05$, compare with control group; #: $p<0.05$, compare with AAC group; &: $p<0.05$, compare with AAC+CYP2J2 group.
